# Risk of Infections Among Contacts of COVID-19 Cases in the Healthcare Setting: Experience of One University Hospital

**DOI:** 10.3390/epidemiologia7010002

**Published:** 2025-12-22

**Authors:** Borislav Tošković, Ljiljana Marković-Denić, Milica Brajković, Igor Nađ, Dimitrije Zdravković, Vladimir Nikolić, COVID-19 Working Group

**Affiliations:** 1 University Clinical Hospital Center Bezanijska Kosa, 11000 Belgrade, Serbia; toskovic.borislav@bkosa.edu.rs (B.T.); brajkovic.milica@bkosa.edu.rs (M.B.); nadj.igor@bkosa.edu.rs (I.N.); 2 Faculty of Medicine, University of Belgrade, 11129 Belgrade, Serbia; 3 Institute of Epidemiology, Faculty of Medicine, University of Belgrade, 11129 Belgrade, Serbia; vladimir.nikolic@med.bg.ac.rs

**Keywords:** COVID-19, HAI, secondary attack rate, risk factors, contact tracing, hospital transmission, SARS-CoV-2

## Abstract

Background/Objectives: The onset of infection in patients in contact with a COVID-19-positive index case in healthcare settings depends on intrinsic factors such as demographic factors, immune status, severity of underlying diseases, and comorbidities. Critical extrinsic factors for transmission, especially in hospitals, are length of exposure and distance. This study aimed to determine the risk factors of COVID-19 infections in contacts of COVID-19 index cases by conducting a prospective cohort study. Methods: The prospective cohort study included 186 index patients with confirmed COVID-19 and their 416 close hospital contacts. All contacts were followed for five days and tested using antigen or RT-PCR assays, with additional follow-up through national registries if discharged earlier. Results: The risk of infection was significantly higher in contacts older than 60 years (*p* = 0.009), in those hospitalised within orthopaedics and haematology departments (*p* < 0.001), and in patients whose bed was located within 1.5 m of the index case (*p* < 0.001). Laboratory findings showed significant associations with lower lymphocytes, glucose and higher potassium and creatinine levels, while other haematological and biochemical parameters did not differ. Hyperkalaemia (RR = 6.2 95%CI = 1.2–32.1 *p* = 0.30) and bed distance ≥ 1.5 m (RR = 0.3 95%CI = 0.2–0.6 *p* < 0.001) demonstrated an independent association with COVID-19 infection among contact patients. Conclusions: To reduce nosocomial transmission from unrecognised COVID-19 reservoirs, patients with electrolyte imbalance and lower levels of blood elements should be placed at a greater distance of 1.5 m from others, especially patients in haematology departments.

## 1. Introduction

The COVID-19 pandemic has raised concerns relating to social isolation, economic instability and uncertainty about the virus and its spread. It has also raised many challenges in the hospital, concerning SARS-CoV-2 transmission and the protection of healthcare workers (HCWs) and patients [1,2]. The virus is spread from the respiratory mucosa of infected individuals, symptomatic or asymptomatic, to others in community and healthcare settings through respiratory droplets, air and contact [3]. However, not all exposed to the virus will develop an infection, even when they have been in contact with hospitalised COVID-19 patients, even when sharing the same environment. The onset of disease depends on many intrinsic factors, such as demographic factors, immune status, severity of underlying diseases and comorbidities [4], as well as many external factors like length of exposure, distance, etc. [5]. All of the above have a significant effect on the nosocomial transmission of the virus [6,7]. Contact tracing has proven to be a highly effective method in preventing the spread of the COVID-19 disease both in the population and in healthcare facilities [8,9]. This involves identifying individuals who have had contact with an infected individual. The effectiveness of contact tracing in healthcare settings and outbreak prevention relies on the speed of identifying contacts and the thoroughness of the tracing process [10]. The isolation of infected contacts during the incubation period could prevent the spread of COVID-19 to other persons. Moreover, understanding the risk factors of individuals in diverse hospital departments can help healthcare providers to prepare and take timely and specific measures to prevent and control the spread of COVID-19 in healthcare settings. Although numerous studies have assessed the risk of SARS-CoV-2 infection among healthcare workers, far fewer studies have examined transmission between hospitalised patients. This represents an important gap because patient-to-patient spread may occur under conditions that differ significantly from hospital staff exposure. In many hospitals, including ours, multi-bed rooms with limited space between patient beds, as well as a lack of negative-pressure rooms, are unfortunately common. Patients often remain in direct proximity for prolonged periods. When they are unable to wear masks continuously due to their underlying conditions and have reduced mobility, this makes them particularly vulnerable to disease transmission from an undetected index case. Understanding the risk factors for patient-to-patient transmission is therefore essential for designing effective prevention strategies in real-world hospital environments. This study aimed to identify the risk factors for COVID-19 infections among contacts of COVID-19 index cases by conducting a prospective cohort study.

## 2. Materials and Methods

A prospective cohort was conducted at the University Clinical Hospital Center Bezanijska Kosa, Belgrade, Serbia, from 1 January to 31 December 2023. The cohort included 186 patients older than 18 years with confirmed COVID-19 infection (index cases) and their close hospital contacts. All contacts were surveyed five days in accordance with the average incubation period of 3–5 days for the Omicron SARS-CoV-2 variant [11]. The department doctors and nurses regularly examined patients and monitored fever, respiratory symptoms, and other symptoms of the disease.

### 2.1. Testing Policy

During hospitalisation, if a clinical suspicion for SARS-CoV-2 infection existed, the patient was tested with antigen tests (Panbio™ COVID-19 Ag Rapid Test Device, Abbott Rapid Diagnostics Jena GmbH, Orlaweg 1, D-07743 Jena, Germany). In our country, the antigen test has been available since November 2020. A positive test result indicated that an infection was present. In the case of a negative test where the patient had symptoms of the disease, testing with a real-time reverse-transcription polymerase chain reaction (RT-PCR) test was performed on the same day. The PCR test was sent to the reference laboratory of the University Clinical Center of Serbia for further processing. The result was obtained within 12–24 h. In addition, screening for SARS-CoV-2 on admission to our hospital was performed until 6 April 2024, as it was recommended for all hospitals in the country. Since April 2024, screening on admission was stopped due to a small number of COVID-19 cases in the population.

The same IPC team that tested the index case collected the samples for antigen and PCR tests from their contacts on the day of the index case testing positive (day zero), and the fifth day after exposure to the index cases. If the contact patients were discharged before day 5 after exposure index case due to a good status of their underlying disease, they were surveyed by telephone. All patients received written instructions in their discharge documents to contact a physician immediately if respiratory symptoms occurred. The results of eventual COVID-19 testing after discharge were checked in the national database of COVID-positive cases, based on the national identification number and the patient’s social security number 14 days after the last exposure to the index case, which is the maximum incubation period for COVID-19. The probability that a contact developed COVID-19 and was not captured in the database is very low. No patients were excluded due to missing follow-up data.

Therefore, all contacts were divided into two groups: COVID-positive contact and COVID-negative contact.

### 2.2. Diagnosis of COVID-19

Diagnosis of COVID-19 was performed by detection of SARS-CoV-2 RNA by real-time reverse-transcription polymerase chain reaction (RT-PCR) assay or antigen testing using nasopharyngeal swab. All tests were approved by the Serbian Ministry of Health and were performed in the Laboratory of Molecular Microbiology, Institute for Biocides and Medical Ecology, Belgrade. The three different molecular assays for the qualitative detection of SARS-CoV-2 were used: GeneFinder^TM^ COVID-19 Plus RealAmp Kit (OSANG Healthcare Co., Seongnam, Republic of Korea), Sansure Biotech (Sansure Biotech Inc., Changsha, China), and TaqPath^TM^ COVID-19 CE-IVD RT-qPCR Kit (Thermo Fisher Scientific, Waltham, MA, USA), and were interpreted according to the manufacturer’s instructions. In addition to the tests, clinical, radiographic or/and laboratory parameters were also taken into account.

### 2.3. Case Definitions

Index cases were defined as patients in an outbreak who were first identified by the health staff. They were classified as community-acquired (CA-COVID-19) and healthcare-associated cases (HA-COVID-19). CA-COVID-19 is an infection acquired before admission to the hospital or in the first two days of hospitalisation. HA-COVID-19 cases are categorised according to the day of symptom onset (or first positive test for asymptomatic cases), as follows: possible HA-COVID-19—onset on day 3–7; probable HA-COVID-19—onset on day 8–14; and definite HA-COVID-19—onset on day 15 and later, according to the definitions provided by ECDC [12]. Contact case was defined as an individual being in a closed environment (e.g., patient room) for more than 15 min with a confirmed COVID-19 case within 2 days before symptom onset to 10 days after symptom in a case [13]. In our hospital, there are no ventilated rooms with a negative pressure, only natural ventilation. All contacts were required to wear a surgical mask while in the multiple-bed isolation room and when they leave the room for examination or go to the toilet.

### 2.4. Data Collection

Our tertiary-level university hospital was a dedicated facility for COVID-19 from 23 June 2021 to 14 March 2022, and then it returned to its usual operation. The hospital has surgical and internal medicine departments, without a psychiatric ward, and children wards. Epidemiologist and infection control nurses from the department for prevention of healthcare-associated infections (infection prevention and control—IPC team) regularly conduct continuous surveillance of all patients admitted to the hospital. The IPC team promptly tested patients with COVID-19 symptoms using rapid antigen or PCR tests due to their excellent cooperation with doctors in the clinical departments. Concurrently, the IPC team performs contact tracing, collecting detailed information about all patients or healthcare workers who were in close contact with the index case up to two days before the onset of COVID symptoms. Data from patient medical histories, temperature records, and data from the hospital information system were collected. Every confirmed case of COVID-19, as well as all other infectious diseases and healthcare-associated infections, is reported electronically to the Serbian public health system. In addition, it was mandatory for the results of all antigen and PCR tests to be reported to the national COVID database, which also includes data on testing in the primary health centres and laboratories.

Laboratory data collected from the hospital laboratory information system. Data on comorbidity were collected from the hospital information system (HIS). Comorbidity was defined as the presence of one or more additional chronic diseases or conditions in a patient. Hypertension was defined as a patient’s history of using antihypertensive medication. Diagnosis of heart failure was based on symptoms and signs, objective evidence, and biomarkers [14]. Malignancy and pulmonary embolism meant a history of disease in the last five years. All diagnoses were taken from the diagnosis entered in the HIS.

### 2.5. Statistical Analysis

Data were analysed using descriptive and statistical methods. Categorical variables were presented as absolute numbers and percentages, while continuous variables were summarised as medians with minimum and maximum values due to non-normal distributions. Group comparisons between COVID-19 positive and COVID-19 negative contact patients were performed using the Chi-square test or Fisher’s exact test where appropriate for categorical variables, and the Mann–Whitney U test was used for continuous variables.

The secondary attack rate (SAR) was calculated as the proportion of COVID-19 infections among close contacts of index cases, with 95% confidence interval (CI) estimates. Subgroup analyses of SAR were conducted according to the classification of index cases (community-acquired, possible, probable, or definite hospital-acquired COVID-19).

To identify independent predictors of COVID-19 positivity among contact patients, we performed a multivariable logistic regression analysis. Variables considered clinically relevant and showing a *p* value ≤ 0.05 in analyses were included in the initial model. These included age, malignancy, lymphocyte categories, glucose categories, potassium categories, creatinine categories, bed distance from the index case, and hospital ward. Odds ratios (ORs) with 95% confidence intervals (CIs) were calculated. Model fit was assessed using the Hosmer–Lemeshow test, and multicollinearity was evaluated by examining variance inflation factors (VIFs).

A *p* value < 0.05 was considered statistically significant. The statistical analyses were performed using SPSS version 26.0 software (SPSS Inc., Chicago, IL, USA).

### 2.6. Ethics Statement

This study was conducted in accordance with the amended Declaration of Helsinki and was reviewed and approved by the Institutional Board of the University Clinical Hospital Center Bezanijska Kosa (No 542/2022).

## 3. Results

Out of 602 patients who were surveyed during the study period, 186 were COVID-19 index cases. The community-acquired COVID-19 had 90 (48.4%) of them, and the other patients (51.6%) had hospital-acquired COVID-19, resulting in 28.5% possible HA-COVID-19, 17.7% probable HA-COVID-19 and 5.4% definite HA-COVID-19.

There were 416 patients in close contact with these index cases, primarily in the same patient rooms of different hospital departments. A total of 78 patients in contact with index cases developed COVID-19, with 72 testing positive during hospitalisation and 5 after discharge. Another 338 patients remained negative after the average incubation period (5 days) during which they were surveyed. Therefore, the secondary attack rate was 18.8% (95%CI 15.1–22.8), from 7.8 for contact with community-acquired COVID-19, 6.2% for contact with possible HA-COVID-19, 36.3% for contact with probable HA-COVID-19, and 31.7% for contact with definite HA-COVID-19 (*p* < 0.0001).

The main characteristics of COVID-positive and COVID-negative contact patients are presented in Table 1. Although the difference in median age between groups was small (70 vs. 73 years), the categorical comparison (>60 vs. ≤60 years) demonstrated a significant increase in risk (89.7% vs. 76.3%, *p* = 0.009). There was no statistically significant difference regarding the sex of contact COVID-positive and negative cases (*p* = 540), number of comorbidities (*p* = 0.623), and smoking status (*p* = 0.220).

A higher percentage of COVID-positive patients who acquired the infection through contact with index cases were overweight (BMI 25 to 29.9 kg/m^2^). The difference was marginally statistically significant (*p* = 0.056). The presence of other comorbidities such as malignant tumours, COPD, asthma, heart failure, hypertension, diabetes and types of therapy for this disease, pulmonary embolism, did not differ significantly between positive and negative contacts (Table 2).

At index case positivity time, the distribution of laboratory values across reference categories showed no significant differences between COVID-19 positive and negative contacts, except for lymphocytes, glucose, creatinine and potassium (Table 3). In COVID-positive contacts, lower lymphocytes percentage (*p* = 0.005), lower blood glucose level (*p* = 0.044), higher creatinine level (*p* = 0.043), and hyperkalaemia (*p* = 0.010) were observed. All other parameters, including leukocytes, erythrocytes, neutrophils, lymphocytes, liver enzymes, renal markers, coagulation parameters, fibrinogen, showed no significant group differences (all *p* > 0.05).

Patients who were in proximity to the COVID-19 index case in the patient’s room (beds that were <1.5 m away) were more likely to acquire COVID-19 than those who were at a higher distance (>1.5 m) (*p* < 0.001). The COVID-positive patients in contact with index case were significantly more frequently hospitalised in the orthopaedics and haematology departments (*p* < 0.001) (Table 4).

In the multivariable logistic regression model, potassium levels demonstrated an independent association with COVID-19 infection among contact patients (Table 5). Compared with patients who had lower potassium levels, hyperkalaemia was significantly associated with increased risk: patients with elevated potassium had more than a six-fold higher likelihood of infection (RR = 6.2; 95% CI: 1.2–32.1; *p* = 0.030). Bed distance remained a strong independent predictor of infection. Patients whose beds were positioned ≥1.5 m from the index case had significantly lower risk of becoming COVID-19 positive compared with those located at <1.5 m (RR = 0.3; 95% CI: 0.2–0.6; *p* < 0.001).

## 4. Discussion

Our study showed that older and overweight patients, patients with hyperkalaemia, higher creatinine levels, hypoglycaemia, and low lymphocyte levels, are more susceptible to infection when they were in contact with COVID-19 index cases in the hospital. Hospital environment risk factors include the distance of beds from COVID-19 index cases of less than 1.5 m, and hospitalisation in departments with a higher risk of infection (haematology and orthopaedics). Hyperkalaemia was an independent risk factor for secondary COVID-19 cases, while a distance between patient beds of less than 1.5 m was an independent protective factor.

Patients with COVID-19 infection may have electrolyte disorders. Hypokalaemia and hyperkalaemia in COVID-19 patients are both associated with a poor prognosis.

We found that a higher percentage of patients with hyperkalaemia became positive in contact with a COVID-19 index case. It is known that COVID-19 patients with hyperkalaemia have a higher risk of mortality. The virus can affect potassium balance through disruption of renin–angiotensin–aldosterone system (RAAS) and epithelial sodium channels (ENaC) [15]. More pronounced hyperkalaemia is found in individuals who are simultaneously affected by heart failure and another concomitant illness [16]. An abnormally high potassium level in the blood might be an indication of an acid–base imbalance with severe acute respiratory distress syndrome [17]. It highlights the crucial significance of maintaining potassium homeostasis in the clinical progression of these individuals. However, hyperkalaemia is not a typical risk factor for developing an infection itself. It is often an associated condition in patients who are already ill, and it probably makes them more susceptible to other diseases. Among other laboratory findings, increased creatinine level was an independent factor that predicted adverse outcomes in COVID-19 [18]. Together, elevated creatinine and potassium levels are an indicator of renal insufficiency and were found to be predictors of a worse outcome in COVID-19 patients upon their admission to the hospital [19]. To understand how elevated potassium and creatinine levels contribute to COVID-19 development, it is necessary to conduct more studies with greater numbers of participants. A higher percentage of our contact patients had hypoglycaemia. Patients with COVID-19 may develop hypoglycaemia due to the use of some medications against this condition, even after receiving a COVID-19 vaccine. Also, if patients have diabetes mellitus, chronic complications can lead to hypoglycaemia. Therefore, the level of glycaemia should be carefully controlled during the course of the disease [20].

When exposed to an index case, patients with low lymphocytes were more likely to become COVID-positive, as has been demonstrated. This suggests the role of lymphocytes in defending against infections. The production of antibodies and the regulation of the immune response are carried out by these cells. Lymphopenia has been identified as a potential marker and prognostic factor of COVID-19 severity, too [21]. It is possible that, besides well-known, pre-existing comorbidities, changes in laboratory results can be predictors of higher susceptibility to COVID-19 onset in contact with COVID-19 cases. On the other hand, it was revealed that lymphopenia is also a marker of the early undetected SARS-CoV-2 [22,23]. It is possible that in a small number of cases in our study, low lymphocyte levels were the result of still undiagnosed COVID-19.

Contrary to the listed modifiable risk factors, older age is a well-known risk factor for COVID-19, one which clinicians are not able to modify. Our results are consistent with many studies that have shown that older people have a higher risk of developing COVID-19 [24,25]. Our observation that older contacts had a higher likelihood of becoming COVID-19 positive is consistent with global evidence. A recent systematic review reported that secondary attack rates increase with age, reaching the highest levels among individuals ≥65 years [26].

Contact time and contact distance play an important role in the transmission of COVID-19 to persons in contact with the infected person [27]. Based on droplet transmission length, the World Health Organization and European Centre for Disease prevention and Control recommended at least one metre between the edges of beds in multi-bed rooms before and at the end of the COVID-19 pandemic [28,29]. In some countries, the minimum bed space should be 2 m or more [30,31]. Our study has revealed that a distance below 1.5 m increases the risk of SARS-CoV-2 transmission. In addition, the higher risk of infection in patients in haematological wards of our hospital is in accordance with the results of other studies [32]. The higher percentage of infected patients in the orthopaedic ward can be attributed to patient rooms with a larger number of beds and a shorter distance between them in our hospital.

Disease onset depends on intrinsic factors, immunity and severity of the underlying disease, as well as distance from the index case during hospitalisation. In our study, we demonstrated a secondary attack rate of 18.8%. At the beginning of the COVID-19 pandemic, secondary cases were rare if appropriate measures were taken [27]. Later, even in developed countries that had quality personal protective equipment and where compliance with its use was high, secondary case rates were much higher. For example, in one tertiary care centre in Switzerland, the incidence rate of probable or definite healthcare-associated COVID-19 was 7% while the secondary attack rate was 23%, primarily due to the exposure to the large number of presymptomatic or asymptomatic COVID-19 patients [33]. This study revealed that the COVID-19 secondary attack rate in the hospital was significantly lower if patients had contact with community-associated COVID-19 than if they had contact with one of the three types of healthcare-associated COVID-19. It was found that about half of hospital-onset COVID-19 cases caused onward transmission, whereas only 4% of community-acquired cases resulted in onward transmission [34]. According to the results of our study, secondary attack rate was much higher in contact with probable and definite hospital-acquired index cases. This is similar as results from other studies [35]. The classification of an infection as “probable” or “definite” hospital-acquired typically depends on the timing of symptom onset or a positive test relative to the date of admission, 8 days and more after admission. In multi-bed rooms, especially where the change in patients is faster, more of them may come into contact with the index case, and the SAR risk may be higher. The viral load of the index patients is considered a much more significant factor for SARS-CoV-2 transmission than that is the contact time or the proximity of the contact patients to the index in the same room [36].

However, in our hospital, all patients with suspected or confirmed SARS-CoV-2 infection, i.e., index cases, were promptly placed in a separate isolation room, or cohort isolation was organised in case there were multiple patients with similar symptoms simultaneously. According to the national recommendations, which are in line with the WHO guidance, patients in isolation should wear a surgical mask if they have to leave the room for additional examinations, or go to the toilet if the isolation room does not have one [37,38]. Isolation of contacts was either in a single-bed room, although on-site isolation in a multiple-bed room was more often used due to the small number of single rooms and the large number of hospitalised patients. This alternative on-site isolation strategy has been successfully applied in other hospitals [36], although it was demonstrated that COVID-19 incidence is higher in multiple-bed rooms [39]. Unfortunately, there are no negative-pressure rooms in our hospital; only natural ventilation is used. The door of the respiratory isolation room should always be closed. When entering the isolation room, healthcare workers and other staff are required to wear an N95 respirator or another type of respirator, gloves, and an isolation gown. Visiting patients in the isolation room was not allowed. Family members could visit the patient in exceptional circumstances if a poor outcome was expected. They wore personal protective equipment, the same as hospital staff. All of the above can help reduce the risk of SARS-CoV-2 transmission when strictly followed.

The main limitation of our study is the lack of SARS-CoV-2 nucleic acid testing and whole-genome sequencing, which would prove that the same subtype of the SARS-CoV-2 virus was transmitted from the index patient to patients who were in contact with them. According to published data in our country in January 2023, the BA.5.1.12 sub-lineage was the most frequent [40], until the Omicron XBB.1.5 sub-lineage appeared, which quickly spread to all European countries [41]. Possible misclassification of exposure could be a limitation of the study. However, we consider this to be a negligible number. All contacts were tested with antigen and/or PCR tests on day zero and day five from the date of their last contact with the COVID-19 case. If any of the contacts tested positive on the test, their contacts were monitored for the next 5 days, even if they had to be transferred to another hospital ward due to their underlying illness.

A constant challenge in interpreting results is the sensitivity of antigen tests. According to a systematic review and meta-analysis, the sensitivity of antigen tests was estimated to be 69% as of 2022 [42]. Later, as the rate of individuals with typical COVID-19 symptoms decreased, sensitivity decreased as well [43]. In our study, all contacts are regularly tested with an antigen test. If the test was negative and they had COVID-19 symptoms, they were also tested with a PCR test. Our belief is that COVID-19 was only asymptomatic in a very small percentage of our patients in 2023.

One of the advantages of this study is that all patients were followed prospectively, and the same IPC team performed data collection, thereby minimising the possibility of inter-observer variability. Namely, the same assigned IPC team members took nasopharyngeal swabs for both antigen and PCR tests, had insight into the patient’s health status, and reviewed the patient’s medical history and laboratory results with the attending physician. Additionally, the proposal for patient isolation measures was developed by a hospital epidemiologist, and its implementation was monitored by members of the IPC team. Some laboratory categories contained very few patients—for example, the group with elevated lymphocytes, which limits the statistical reliability of estimates de-rived from these subgroups. These lower group sizes reduce power and may produce un-stable effect estimates; therefore, findings in such categories should be interpreted with caution.

## 5. Conclusions

According to our study findings, patients who have contact with COVID-19 are more susceptible to the disease if they have an electrolyte imbalance, lower blood element levels, and a shorter distance from a COVID-19 patient. To reduce nosocomial transmission from unrecognised COVID-19 reservoirs, patients with these laboratory anomalies should be separated from others by 1.5 m particularly those in haematology departments.

## Figures and Tables

**Table 1:** Characteristics of patients in contact with COVID-19 index cases, 2023.

	Total n (%)	COVID- Positive Contacts n (%)	COVID- Negative Contacts n (%)	*p* Value
Total	416 (100.0)	78 (18.8)	338 (81.3)	
Sex				
Male	211 (50.7)	169 (50.0)	42 (53.8)	0.540
Female	205 (49.3)	169 (50.0)	36 (46.2)	
Age (years)—median (min–max)	71 (18–98)	70 (18–98)	73 (19–92)	0.076
≤60	88 (21.2)	8 (10.3)	80 (23.7)	**0.009**
>60	328 (78.8)	70 (89.7)	258 (76.3)	
Number of comorbidities				
0	45 (11.1)	7 (9.1)	38 (11.6)	0.623
1	121 (30.0)	21 (27.3)	100 (30.6)	
≥2	238 (58.9)	49 (63.6)	189 (57.8)	
Smoking status				
Nonsmoker	160 (60.6)	131 (60.4)	29 (61.7)	0.220
Current smoker	80 (30.3)	69 (31.8)	11 (23.4)	
Former smoker	24 (9.1)	17 (7.8)	7 (14.9)	

Bold is used to highlight statistical significance.

**Table 2:** Comorbidities of patients in contact with COVID-19 index cases.

	Total n (%)	COVID- Positive Contacts n (%)	COVID- Negative Contacts n (%)	*p* Value
BMI, kg/m^2^	26.0 (15.6–41.5)	26.2 (18.6–35.6)	25.5 (15.6–41.5)	0.654
BMI categories, kg/m^2^				
Normal weight (<25)	60 (45.8)	8 (33.3)	52 (48.6)	**0.056**
Overweight (25 to29.9)	39 (29.8)	12 (50.0)	27 (25.2)	
Obese (≥30)	32 (24.4)	4 (16.7)	28 (26.2)	
Comorbidities				
Malignancy	100 (24.0)	76 (22.5)	24 (30.8)	0.123
COPD	69 (16.6)	58 (17.2)	11 (14.1)	0.506
Asthma	17 (4.1)	12 (3.6)	5 (6.4)	0.336
Heart failure	115 (27.8)	94 (28.0)	21 (26.9)	0.852
Hypertension	302 (72.8)	245 (72.7)	57 (73.1)	0.946
Diabetes	113 (27.3)	87 (25.9)	26 (33.3)	0.184
Type of therapy for diabetes				
Oral	73 (42.2)	58 (42.0)	15 (31.4)	0.721
Insulin	25 (14.5)	18 (13.0)	7 (20.0)	
Combine	12 (6.9)	10 (7.2)	2 (5.7)	
Pulmonary embolism	29 (7.1)	25 (7.6)	4 (5.2)	0.465

BMI—body mass index; COPD—chronic obstructive pulmonary diseases. Bold is used to highlight statistical significance.

**Table 3:** Haematological and biochemical parameters of patients in contact with COVID-19 index cases at index positivity time.

	Total Median (Min–Max)	COVID- Positive Contacts n (%)	COVID- Negative Contacts n (%)	*p* Value
Leukocytes WBC [white blood cells]	9.7 (0.6–96.1)	10.2 (0.6–68.7)	9.6 (0.8–96.1)	0.282
Erythrocytes RBC [red blood cells]	4.2 (1.0–7.0)	4.2 (1.0–5.8)	4.2 (1.3–7.0)	0.882
Neutrophiles (%)	76.2 (3.0–96.9)	75.1 (9.7–94.5)	76.3 (3.0–96.9)	0.337
Neutrophiles (n)	7.2 (0.1–261.0)	7.4 (0.2–21.4)	7.2 (0.1–261.0)	0.547
Lymphocytes (1–4 × 10^9^/L)	1.4 (0.2–203.0)	1.4 (0.2–26.9)	1.3 (0.2–203.0)	0.223
Lymphocytes categories (%)				**0.005**
Lower (<1)	100 (32.6)	24 (40.0)	76 (30.8)	
Normal (1–4)	205 (66.8)	34 (56.7)	171 (69.2)	
Higher (>4)	2 (0.7)	2 (3.3)	0 (0.0)	
NLR	5.0 (0.02–208.8)	4.7 (0.1–37.5)	5.0 (0.02–208.8)	0.330
Urea (mmol/L)	7.4 (1.7–40.8)	7.3 (3.7–39.2)	7.4 (1.7–40.8)	0.529
Glucose	6.7 (2.8–38.4)	6.4 (2.8–38.4)	6.8 (2.8–33.9)	0.507
Glucose categories (%)				**0.044**
Lower	7 (1.9)	4 (5.4)	3 (1.0)	
Normal (4.1–5.9)	119 (31.9)	23 (31.1)	96 (32.1)	
Higher	247 (66.2)	47 (63.5)	200 (66.9)	
Enzymes				
AST (U/L) [aspartate aminotransferase]	22.0 (0.8–1037.0)	21.0 (5.5–311.0)	22.0 (0.8–1037.0)	0.612
ALT (U/L) [alanine aminotransferase]	21.0 (0.5–1383.0)	20.0 (5.0–1383.0)	21.0 (0.5–499.0)	0.250
LDH (U/L) [lactate dehydrogenase]	420.5 (13.0–1999.0)	417.0 (13.0–1586.0)	424.0 (86.0–1999.0)	0.990
Troponin (ng/L)	35.5 (0.3–25,000.0)	25.0 (0.3–7166.0)	36.0 (0.5–25,000.0)	0.812
K (mmol/L)	4.3 (2.6–7.2)	4.3 (3.1–6.4)	4.2 (2.6–7.2)	0.062
K categories (%)				**0.010**
Lower	23 (5.9)	3 (4.1)	20 (6.3)	
Normal (3.5–5.1)	238 (84.3)	56 (76.7)	272 (86.1)	
Higher	38 (9.8)	14 (19.2)	24 (7.6)	
Na (mmol/L)	139.0 (116.0–161.0)	138.0 (120.0–149.0)	139.0 (116.0–161.0)	0.376
Cl (mmol/L)	102.0 (78.6–130.1)	102.0 (78.6–116.0)	102.0 (83.9–130.1)	0.807
Creatinine (μmol/L)	92.0 (34.0–491.0)	96.5 (50.0–491.0)	91.0 (34.0–384.0)	0.245
Creatinine categories (%)				**0.043**
Lower	31 (11.6)	1 (1.9)	30 (14.1)	
Normal (M: 62–115; F: 49–90)	158 (59.2)	36 (66.7)	122 (57.3)	
Higher	78 (29.2)	17 (31.5)	61 (28.6)	
Proteins (g/L)	68.0 (41.0–98.0)	67.0 (53.0–81.0)	68.0 (41.0–98.0)	0.666
Albumins (g/L)	39.0 (20.0–52.0)	40.0 (24.0–48.0)	39.0 (20.0–52.0)	0.795
CRP (mg/L)	20.7 (0.5–623.7)	21.2 (0.5–273.8)	19.5 (0.5–623.7)	0.929
INR	1.1 (0.8–8.6)	1.1 (0.9–6.8)	1.1 (0.8–8.6)	0.150
Fibrinogen	3.9 (0.5–25.5)	3.9 (1.2–11.4)	3.9 (0.5–25.5)	0.861

NLR—neutrophil-to-lymphocyte ratio; INR—prothrombin time; CRP—C-reactive protein. Bold is used to highlight statistical significance.

**Table 4:** Epidemiological characteristics of patients in contact with COVID-19 index cases.

	Total n (%)	COVID- Positive Contacts n (%)	COVID- Negative Contacts n (%)	*p* Value
Bed distance				
<1.5 m	104 (25.1)	36 (46.2)	68 (20.2)	**<0.001**
≥1.5 m	310 (74.9)	42 (53.8)	268 (79.8)	
Hospital wards				**<0.001**
ICU	60 (14.4)	12 (15.4)	48 (14.2)	
Surgery	70 (16.8)	12 (15.4)	58 (17.2)	
Orthopaedy	44 (10.6)	30 (38.5)	14 (4.1)	
Haematology	38 (9.1)	10 (12.8)	28 (8.3)	
Other internal medicine wards	204 (49.0)	14 (17.9)	190 (56.2)	
Total	416 (100.0)	78 (100.0)	338 (100.0)	

ICU—Intensive care unit. Bold is used to highlight statistical significance.

**Table 5:** Multivariable logistic regression for identifying independent risk factors for contact COVID-19 positivity.

	RR	95% Confidence Interval	*p* Value
Age (years)			
≤60			
>60			
Lymphocytes categories			
Lower (<1)			
Normal (1–4)			
Higher (>4)			
Glucose categories			
Lower			
Normal (4.1–5.9)			
Higher			
K categories			
Lower	Ref.		
Normal (3.5–5.1)	2.2	0.5–10.2	0.295
Higher	**6.2**	**1.2–32.1**	**0.030**
Creatinine categories			
Lower			
Normal (M:62–115; F:49–90)			
Higher			
Bed distance			
<1.5 m			
≥1.5 m	**0.3**	**0.2–0.6**	**<0.001**
Hospital wards			
ICU			
Surgery			
Orthopaedy			
Haematology			
Other internal medicine wards			
Total			

RR—risk ratio; ICU—Intensive care unit. Bold is used to highlight statistical significance.

## Data Availability

The data presented in this study are available on request from the corresponding author due to (privacy).

## Abbreviations

The following abbreviations are used in this manuscript:

ALT	Alanine Aminotransferase
AST	Aspartate Aminotransferase
BMI	Body Mass index
CA-COVID-19	Community-Acquired COVID-19
Cl	Chloride
COPD	Chronic Obstructive Pulmonary Disease
COVID-19	Coronavirus Disease 2019
CRP	C-Reactive Protein
ECDC	European Centre for Disease Prevention and Control
HA-COVID-19	Healthcare-Associated COVID-19
ICU	Intensive Care Unit
INR	International Normalised Ratio
IPC	Infection Prevention and Control
LDH	Lactate Dehydrogenase
NLR	Neutrophil-to-Lymphocyte Ratio
PCR	Polymerase Chain Reaction
RBC	Red Blood Cells
RT-PCR	Reverse-Transcription Polymerase Chain Reaction
SAR	Secondary Attack Rate
SARS-CoV-2	Severe Acute Respiratory Syndrome Coronavirus 2
SPSS	Statistical Package for the Social Sciences
WBC	White Blood Cells
